# Structural Magnetic Resonance Imaging Can Identify Trigeminal System Abnormalities in Classical Trigeminal Neuralgia

**DOI:** 10.3389/fnana.2016.00095

**Published:** 2016-10-19

**Authors:** Danielle D. DeSouza, Mojgan Hodaie, Karen D. Davis

**Affiliations:** ^1^Department of Neurology and Neurological Sciences, Stanford UniversityStanford, CA, USA; ^2^Division of Brain, Imaging and Behavior-Systems Neuroscience, Krembil Research Institute, University Health NetworkToronto, ON, Canada; ^3^Institute of Medical Science, University of TorontoToronto, ON, Canada; ^4^Division of Neurosurgery, Toronto Western Hospital and Department of Surgery, University of TorontoToronto, ON, Canada

**Keywords:** trigeminal neuralgia, structural MRI, DTI, plasticity, pain

## Abstract

Classical trigeminal neuralgia (TN) is a chronic pain disorder that has been described as one of the most severe pains one can suffer. The most prevalent theory of TN etiology is that the trigeminal nerve is compressed at the root entry zone (REZ) by blood vessels. However, there is significant evidence showing a lack of neurovascular compression (NVC) for many cases of classical TN. Furthermore, a considerable number of patients who are asymptomatic have MR evidence of NVC. Since there is no validated animal model that reproduces the clinical features of TN, our understanding of TN pathology mainly comes from biopsy studies that have limitations. Sophisticated structural MRI techniques including diffusion tensor imaging provide new opportunities to assess the trigeminal nerves and CNS to provide insight into TN etiology and pathogenesis. Specifically, studies have used high-resolution structural MRI methods to visualize patterns of trigeminal nerve-vessel relationships and to detect subtle pathological features at the trigeminal REZ. Structural MRI has also identified CNS abnormalities in cortical and subcortical gray matter and white matter and demonstrated that effective neurosurgical treatment for TN is associated with a reversal of specific nerve and brain abnormalities. In conclusion, this review highlights the advanced structural neuroimaging methods that are valuable tools to assess the trigeminal system in TN and may inform our current understanding of TN pathology. These methods may in the future have clinical utility for the development of neuroimaging-based biomarkers of TN.

Trigeminal neuralgia (TN), characterized by excruciating attacks of electric shock-like unilateral facial pain, has been described as one of the most severe pains one can experience. There are an estimated 15,000 new cases of TN diagnosed in the United States each year and with greater awareness and better diagnostic criteria, recent studies suggest that this is likely an underestimate (Pollock and Ecker, 2005; Hall et al., 2006). Each year, thousands of patients undergo surgery for TN in the United States, with estimated costs exceeding $100 million (Pollock and Ecker, 2005). These treatments result in excellent outcomes for many patients, but some patients do not experience any pain relief. As such, noninvasive measures to predict clinical outcomes from treatment are needed. Our understanding of TN has drastically grown over the past few centuries, but the etiology and pathophysiological mechanisms underlying TN are still not well understood. Recent studies using noninvasive neuroimaging methods, such as structural MRI, have provided new insight into our understanding of TN and mechanisms of treatment response. In this review, we will discuss theories of TN pathophysiology, how advanced neuroimaging methods contribute to our current understanding of TN, and future directions for clinical trials that may use these methods to inform treatment selection and response for TN patients based on brain and/or nerve biomarkers.

## Central and Peripheral Theories of TN Etiology and Pathophysiology

In the 1850s, some theorized that TN might involve a central etiology. For example, Trousseau observed that because the painful paroxysms experienced by TN patients resembled a convulsive seizure he termed “epileptiform neuralgia” (Trousseau, 1853), TN was due to paroxysmal activity in the trigeminal system similar to cerebral paroxysmal discharges experienced by epilepsy patients. Other evidence suggesting a CNS contribution to TN was that pain attacks were self-sustained once triggered, larger in magnitude than the stimulus, and were followed by a refractory period during which time another attack could not be triggered (Kugelberg and Lindblom, 1959). As such, the antiepileptic medication, carbamazepine was considered as a treatment for TN, and is still one of the most frequently prescribed medications for TN (Hall et al., 2006). Antiepileptic medications can effectively manage TN pain initially and not only act on the brain but also on peripheral nerves by slowing motor and sensory fiber conduction velocities (Traccis et al., 1983).

We now know that central sensitization or use-dependent synaptic plasticity can occur when there is the sustained or repetitive activation of primary afferent fibers including nociceptors (Woolf and Salter, 2000). CNS neurons (e.g., those in the dorsal horn of the spinal cord or in the spinal trigeminal nucleus of the brainstem) change the gain of their responsiveness to sensory input such that they have amplified responses to noxious and innocuous stimuli. This change is triggered by intracellular cascades, which lead to facilitated excitatory transmission and depressed inhibition (Woolf and Salter, 2000). When there is inflammation, neuropeptides and neurotrophic factors such as substance P and brain-derived neurotropic factor are released from the terminals of the primary afferents (Kidd and Urban, 2001). This activates intracellular cascades with the net result being facilitated excitatory transmission produced by an exaggerated response to stimuli, an expansion of the receptive field size such that there is a spread of sensitivity to regions outside of the injured tissue, and a reduction in the threshold for activation of afferent inputs so that central neurons are responsive to even innocuous inputs (Kilo et al., 1994; Woolf and Salter, 2000; Kidd and Urban, 2001). There is evidence suggesting that many of these changes are mediated by N-methyl-D-aspartate (NMDA) receptors. There are also NMDA receptor-independent mechanisms for facilitating excitatory synaptic transmission, primarily via mechanisms involving a subset of α-amino-3-hydroxy-5-methyl-4-isoxazolepropionic acid (AMPA) receptors (Gu et al., 1996). Furthermore, there can be a long-term depression of gamma-aminobutyric acid (GABA) and glycine dorsal horn neurons, which have inhibitory effects on afferent input (Sandkuhler and Gebhart, 1984; Woolf and Salter, 2000).

**Figure 1:**
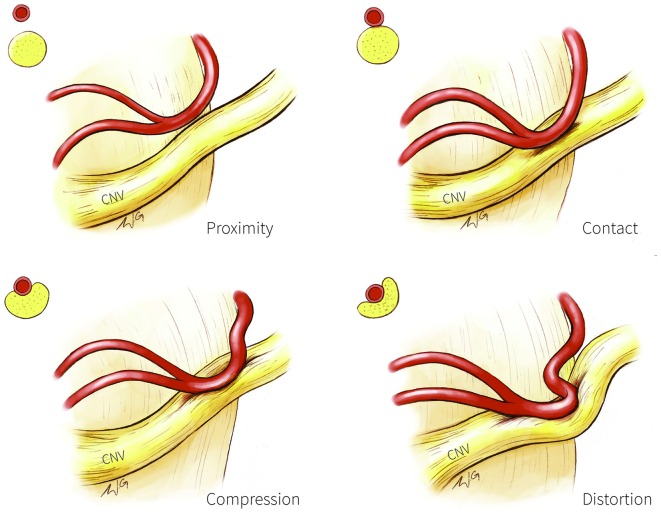
**Trigeminal nerve-vessel relationships.** A schematic illustration depicting examples of trigeminal-nerve vessel relationships at the cerebellopontine angle, with corresponding cross-sections in the upper left corner of each panel. Proximity (upper left panel): vessel in close proximity to trigeminal nerve but without contact; Contact (upper right panel): simple contact of the trigeminal nerve by a vessel but without compression or distortion of the nerve; Compression (bottom left panel): vessel compressing the trigeminal nerve; Distortion (bottom right panel): compression severe enough to deform the trigeminal nerve. CN V, trigeminal nerve.

Central contributions in TN remain debatable, but ample evidence, primarily from surgical experience, suggests that the origin of TN and its pathogenesis involves the trigeminal nerve at the root entry zone (REZ; Nurmikko and Eldridge, 2001). The most common theory of classical TN etiology is a peripheral theory involving the compression of the trigeminal REZ by blood vessels (Nurmikko and Eldridge, 2001). This was first described by Dandy (1929), who observed that TN patients frequently had grooves or indentations in their trigeminal nerves made by offending vessels (Figure 1). Several lines of evidence have led to the proposition that over time this sustained static or pulsatile vascular compression leads to focal points of damage on the trigeminal nerve, resulting in the characteristic pain attacks in TN. Corroborating this, several studies have used ultrastructural and immunohistochemical techniques to demonstrate pathological changes, most notably demyelination of the trigeminal nerves in patients with TN (Kerr and Miller, 1966; Hilton et al., 1994; Rappaport et al., 1997; Love et al., 1998; Devor et al., 2002b; Marinković et al., 2009). Indeed, trigeminal root biopsies from patients with TN show that in regions with the most severe neurovascular compression (NVC) there are few axons remaining and that nearly all of these axons were demyelinated and in close apposition to each other (Rappaport et al., 1997; Devor et al., 2002b). Since we currently lack a validated animal model that reproduces the clinical features of TN, much of what we know about TN pathology comes from these biopsy studies, which limits our ability to test theories of TN pathophysiology.

Compressive syndromes that affect the spinal dorsal REZ (e.g., disc herniation, spinal stenosis and tumor growth) can also be a source of severe pain (Kobayashi et al., 2004). Anatomically, the spinal dorsal REZ not only includes proximal dorsal nerve rootlets, but also Lissauer’s tract and the superficial dorsal horn laminae I-V (Bozkurt et al., 2012). Therefore, pain symptoms can vary depending on the location and extent of the compression in the spinal dorsal REZ. An example can be seen with patients who have spinal disc herniation. When compression affects the dorsal roots of the lumbar spine, radicular pain such as sciatica is common. Pain symptoms of sciatica are variable but like TN pain, it is typically unilateral and can be electric-shock like in quality (Freynhagen and Baron, 2009). However, unlike TN pain, sciatica pain is more frequently persistent and other sensory abnormalities such as numbness and paresthesias are often evident. A minority of TN patients have also reported sensory abnormalities and autonomic symptoms. In one study, 31% of patients reported ipsilateral autonomic symptoms (e.g., conjunctival injection/tearing, running/clogged nose) during pain attacks and for 5% of these patients (*n* = 8), the symptoms occurred with every pain attack (Maarbjerg et al., 2014). These symptoms occurred more commonly in patients that had pain affecting the ophthalmic branch of the trigeminal nerve. In addition, three of these patients had a dual diagnosis of TN and either short-lasting unilateral neuralgiform headache attacks with cranial autonomic features (SUNA), short-lasting unilateral neuralgiform headache attacks with conjunctival injection and tearing (SUNCT), or cluster headache, which may have contributed to these autonomic symptoms.

One theory that has gained recognition for blending pathological findings associated with NVC of the trigeminal REZ and the unique symptoms of TN pain is the *ignition hypothesis* (Rappaport and Devor, 1994) which was more recently updated to account for advances made in our understanding of nerve pathophysiology (Devor et al., 2002a). This theory proposes that TN pain results from an abnormal generation of sensory impulses of trigeminal fibers generated from regions of demyelination, or other structural abnormalities, referred to as pacemaker sites. While damaged neurons can be spontaneously active following injury (Burchiel, 1980; Love and Coakham, 2001; Devor et al., 2002a), other neurons may be silent, but have hair-trigger thresholds such that a momentary stimulation can produce firing lasting several seconds. This phenomenon is referred to as *afterdischarge* (Devor et al., 2002a). Afterdischarge can recruit neighboring neurons via neuron-to-neuron coupling, which this hypothesis suggests may underlie the paroxysmal explosion of pain characteristic of TN. The ignition hypothesis also describes how the activation of the NVC-induced demyelinated fibers that primarily convey non-nociceptive sensory information may result in a TN pain attack. In cases where there is nerve damage such as demyelination, the axons of sensory neurons come in closer contact with each other at these sites, and when one neuron fires, it can now depolarize previously silent neighbors (Rappaport and Devor, 1994; Devor et al., 2002a). When this cross-excitation occurs between individual axon pairs that become electrically coupled, it is referred to as ephaptic crosstalk/transmission (Seltzer and Devor, 1979). In the TN literature, ephaptic crosstalk between fibers mediating light touch (Aβ fibers) and those involved in nociception (Aδ and c fibers), has been considered to account for TN pain attacks that are triggered by brief stimuli such as light touch to the face (Love and Coakham, 2001; Nurmikko and Eldridge, 2001). Corroborating this, electrophysiological recordings in the rat dorsal root ganglion demonstrated that neuronal activity triggered in A-fibers produce a transient depolarization in passive neighboring C-fibers and that approximately 90% of the neurons sampled demonstrate cross-depolarization (Amir and Devor, 2000). The authors concluded that this crosstalk might augment neuropathic sensory abnormalities including pain in the event of nerve injury.

Despite the considerable evidence for TN being a peripheral disorder, some have argued that the NVC theory of TN cannot sufficiently explain the disorder because some individuals can develop TN in the absence of NVC, and some individuals with NVC never develop TN. Additionally, in TN cases where NVC is evident, the CNS may also contribute to TN pathophysiology, since it is well known that CNS plasticity can occur following peripheral nerve injury (Taylor et al., 2009; Davis et al., 2011). For example, peripheral nerve injury can result in the sustained or repetitive activation of primary afferent fibers and central sensitization. This change is triggered by intracellular cascades, which lead to facilitated excitatory transmission and depressed inhibition (Woolf and Salter, 2000). This can occur at one synapse (homosynaptic) or at multiple synapses (heterosynaptic). Classically, TN is characterized by paroxysmal pain with patients typically being pain-free between attacks. Over time, however, these attacks may occur more frequently, with less pain-free periods in between them. It is possible that the CNS changes that accompany peripheral nerve injury contribute to evolving TN symptoms. However, this relationship remains to be determined. While some of the mechanisms underlying TN pathophysiology remain controversial, neuroimaging methods and in particular, studies using MRI to examine trigeminal nerve and brain structure, have provided valuable insight into these mechanisms.

## Structural MRI: A Noninvasive Means to Study Peripheral Theories of TN

Structural MRI is widely used in both clinical practice and research. However, MRI for research typically involves specialized protocols and post-processing techniques. Structural MRI for TN research uses *high-resolution* *anatomical imaging* (variations of T1- and T2-weighted images) to assess anatomical characteristics and patterns of NVC, brain gray matter volume and cortical thickness (CT) and *diffusion imaging* (diffusion tensor images (DTI)) to assess brain white matter and trigeminal nerve microstructure.

Structural MRI can be used to test whether NVC sufficiently explains TN etiology. Some have argued that NVC cannot be the only cause of TN because blood vessels are often seen in the vicinity of the trigeminal nerve on routine autopsy or even on conventional MRI scans of unaffected nerves or in asymptomatic individuals without any TN (Nurmikko and Eldridge, 2001; Miller et al., 2009; Satoh et al., 2009; Antonini et al., 2014). Still, surgical cases suggest approximately 80–90% of TN patients have NVC at their trigeminal nerves ipsilateral to their side of pain (Love and Coakham, 2001; Satoh et al., 2009). But, conventional two-dimensional imaging is not always reliable in detecting NVC (Meaney et al., 1995). To better characterize the anatomical patterns of NVC in patients with TN, high resolution anatomical scans, combined with other non-conventional methods (e.g., three-dimensional time-of-flight MR angiography, spoiled gradient-recalled imaging, nerve/vessel model reconstructions) have shown that NVC at the unaffected trigeminal nerve is typically not as severe as at the affected nerve, nor is it enough to produce nerve dislocation or distortion (Anderson et al., 2006; Lorenzoni et al., 2012). In contrast, nerve dislocation or distortion occurs frequently at the affected trigeminal nerve, suggesting that NVC associated with TN is more severe than at the unaffected nerve or in asymptomatic individuals (Lorenzoni et al., 2012).

Structural MR has also been used to categorize NVC into four categories based on the extent of nerve circumference in contact with the vessel as a proxy of severity: severe (NVC with the vessel covering >20% of the trigeminal nerve circumference); moderate (NVC with vessel covering <20% of the nerve circumference); simple (vessel contacts the nerve; Figure 1, top right) and none (vessel in proximity to the nerve; Figure 1, top left; Satoh et al., 2009). Moderate and severe NVC may result in compression or distortion of the trigeminal nerve (Figure 1, bottom panels). NVC of the affected trigeminal nerve tends to be severe in patients with TN, whereas NVC at the unaffected nerves of patients or in healthy individuals are mostly of the simple contact form (Satoh et al., 2009).

TN is also associated with atrophy of the affected trigeminal nerve (Erbay et al., 2006; Antonini et al., 2014; Leal et al., 2014). High-resolution MR has demonstrated that the mean diameter and cross-sectional area of the trigeminal nerve on the symptomatic side of TN patients is smaller compared to the asymptomatic side. It was proposed that atrophy is likely secondary to structural abnormalities of the trigeminal nerve, including axonal loss and demyelination that result from NVC (Erbay et al., 2006). Corroborating these findings, a recent study reported that trigeminal NVC as detected by MRI is likely to be symptomatic when it is accompanied by anatomical nerve changes including nerve atrophy (Antonini et al., 2014).

Peripheral theories of TN also emphasize the importance of the REZ in TN etiology and pathophysiology, with recent DTI studies providing support of this theory. Anatomically, myelinated axons at the REZ are primarily associated with CNS myelin produced by oligodendrocytes, which transitions to peripheral myelin produced by Schwann cells (Peker et al., 2006). Immunohistochemical studies have demonstrated NVC-induced focal demyelination that largely affects the CNS myelin at the trigeminal REZ (Marinković et al., 2009). In this study, trigeminal nerve roots of patients with trigeminal pain syndromes were obtained during autopsy. Of the patients with TN, 83% had neurovascular relationships at the transition zone between central and peripheral myelin. At this location, various changes in the central myelin were observed including deformation and demyelination. In contrast, segmental demyelination affected only a few individual nerve fibers in the regions adjacent to the NVC (Marinković et al., 2009). The notion that CNS myelin is primarily disrupted at the REZ of TN patients is consistent with peripheral myelin being significantly more resistant to compression and damage. Therefore, the REZ is likely a key region in TN pathophysiology. This provides the rationale for why vascular contact more distally along the trigeminal nerve can occur in individuals without any history of TN (Nurmikko and Eldridge, 2001).

**Figure 2:**
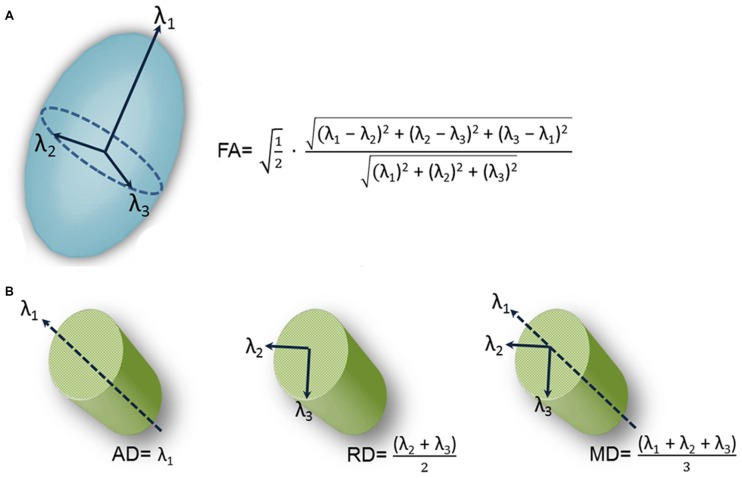
**Schematic representation of diffusion tensor images (DTI)-derived metrics.** A schematic representation of the tensor model and formulas of four DTI-derived metrics: fractional anisotropy (FA; **A**) and axial diffusivity (AD; diffusion along the length of the axon (λ_1_, left), radial diffusivity (RD; diffusion perpendicular to the length of the axon (average of λ_2_ and λ_3_, center) and mean diffusivity (MD; average diffusion regardless of direction (mean of λ_1_, λ_2_ and λ_3_, right; **B**).Figure is adapted from DeSouza et al. (2014) with permission (Rightslink license number: 3823931416268).

Immunohistochemical studies rely on trigeminal nerve biopsy samples extracted during surgical procedures for TN or during autopsy. However, DTI provides a noninvasive means for examining trigeminal nerve microstructure *in vivo*. DTI involves fitting a tensor, which is an ellipsoid shaped mathematical model, at each brain voxel of a diffusion MR scan. The tensor is characterized by three orthogonal eigenvectors and their associated eigenvalues (λ_1_, λ_2_, λ_3_). In general, the shape of the tensor carries information about the three-dimensional character of the water molecules’ diffusion. There are several measures of tissue microstructure that can be derived from these eigenvalues, the most widely used being fractional anisotropy (FA; Figure 2A). FA ranges from zero (i.e., completely isotropic) to one (i.e., completely anisotropic). Diffusion varies for different tissue types because the structural barriers innate to certain tissues hinder diffusion. These variations are reflected by the shape of the tensor derived from a voxel of that tissue type. For example, the shape of a tensor derived from a voxel within the CSF would be roughly spherical in shape, indicating that diffusion is not hindered and molecules can flow equally in all directions. In this case, diffusion is said to be isotropic. In contrast, there are several barriers to diffusion in white matter bundles making diffusion along the length of the axis greater than across it. Diffusion is said to be anisotropic when this occurs and the shape of the tensor is less spherical.

This has motivated a handful of studies to examine structural abnormalities in the trigeminal nerves of patients with TN using FA as measure (Herweh et al., 2007; Fujiwara et al., 2011; Leal et al., 2011; Lutz et al., 2011). In these studies, FA was extracted from the trigeminal nerves of patients and healthy control participants, with the majority of the studies (Herweh et al., 2007; Leal et al., 2011; Lutz et al., 2011) focusing on trigeminal REZ values. The results indicated that FA was lower in the affected REZ of TN patients with NVC, consistent with there being disrupted nerve organization at this location. These studies demonstrated that a noninvasive *in vivo* measure of trigeminal nerve microstructure could be used to detect abnormalities in the trigeminal nerves of TN patients. However, the studies did have some limitations. First, the data were acquired using resolutions that may not have been sufficient to reveal all abnormalities. In DTI, multiple images are collected so that the signal can be sensitized to diffusion in different directions, building up multiple measurements for each voxel in the brain (Johansen-Berg and Rushworth, 2009). In these aforementioned studies, a relatively low number of diffusion gradient-directions were used (i.e., two of these studies collected diffusion data with only six directions; Herweh et al., 2007; Fujiwara et al., 2011), one study with 15 directions (Lutz et al., 2011), and the other with 32 directions (Leal et al., 2011). While only six noncollinear directions are required to estimate the diffusion tensor, many more images are usually required to boost the signal intensity-to-noise ratio (Mukherjee et al., 2008). The rationale for sampling more directions is that it reduces orientational dependence and increases the accuracy and precision of estimating tensor-derived parameters such as FA (Mukherjee et al., 2008). Second, many of these studies primarily assessed the DTI metric FA, that does not fully describe the tensor shape. Therefore, information important for a full interpretation of the findings was limited because other DTI metrics such as mean, radial and axial diffusivities (MD, RD, AD) were not collected. As shown in Figure 2B, AD corresponds to the main diffusion direction, or diffusion along the length of the axon (λ_1_), RD corresponds to diffusion perpendicular to the main diffusion direction (average of λ_2_ and λ_3_), and MD measures the local magnitude of diffusion regardless of direction (average of λ_1_, λ_2_ and λ_3_). These other measures of tissue microstructure have been linked to specific pathophysiological mechanisms such as demyelination, neuroinflammation and edema (Alexander et al., 2007), as diffusion is particularly sensitive to changes in the architecture of cellular membranes that can occur under certain pathological conditions. While reduced FA has been reported across a broad spectrum of disorders (Alexander et al., 2007), the direction of MD, RD and AD changes are more variable depending on the precise underlying pathophysiology and can provide useful information as to why FA is decreased in TN.

**Figure 3:**
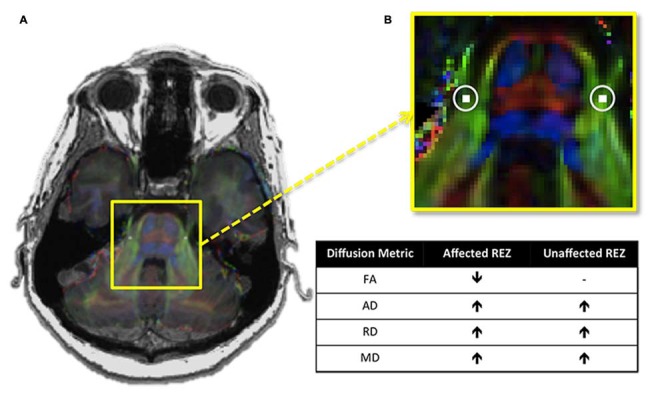
**Trigeminal root entry zone (REZ) abnormalities in trigeminal neuralgia (TN). (A)** An axial T1-weighted MRI slice at the level of the pons is shown on the left, with a DTI image at the same level superimposed over it. The yellow box highlights the pons of the brainstem and the cisternal portions of the left and right trigeminal nerves. **(B)** A zoomed-in version of this box reveals the location of the REZ masks (white squares, highlighted with circles) used to extract each of the four examined DTI metrics in TN patients and controls. Between-group comparisons revealed that TN patients had abnormalities in all four metrics examined. The chart shows the direction of these abnormalities in patients relative to controls. Specifically, TN patients had lower FA at the affected trigeminal REZ, and higher AD, RD and MD bilaterally. DTI, diffusion tensor imaging; FA, fractional anisotropy; RD, radial diffusivity; MD, mean diffusivity; AD, axial diffusivity. − Signifies no significant difference. Panels **(A,B)** were adapted from DeSouza et al. (2014) with permission (RightsLink license number: 3823931416268).

Recently, we assessed all four of these described diffusion metrics at the trigeminal REZ and demonstrated that TN patients not only have lower FA at their affected REZ, but also higher AD, RD and MD (DeSouza et al., 2014; Figure 3). Our finding of increased MD and RD may be linked to NVC-induced focal demyelination of the trigeminal REZ (Marinković et al., 2010), neuroinflammatory processes, and/or edema (Beaulieu, 2002) affecting the trigeminal system, since these increases were also observed at the unaffected REZ. Therefore, DTI can detect subtle pathological features at the trigeminal REZ supporting a role for this region’s involvement in TN pathophysiology.

## Structural MRI Reveals Brain Abnormalities in TN

Brain gray matter abnormalities have been identified in patients with chronic pain (Davis and Moayedi, 2013). The most common location of gray matter abnormalities include regions implicated in the multidimensional experience of pain such as the prefrontal cortex (PFC), insula, anterior and mid-cingulate cortices (ACC, MCC), thalamus, primary and secondary somatosensory cortices (S1, S2), basal ganglia, amygdala and brainstem (Davis and Moayedi, 2013). These abnormalities are commonly found for many types of chronic pain including those affecting the trigeminal system such as migraine (Rocca et al., 2006), trigeminal neuropathic pain (DaSilva et al., 2008; Gustin et al., 2011), and temporomandibular disorder (TMD; Moayedi et al., 2011). Since some abnormalities are common across most chronic pains (e.g., cortical thinning in the anterior insula, cingulate cortex and dorsolateral prefrontal cortices; Davis and Moayedi, 2013), these areas likely reflect pain chronicity in general, and may be associated with negative affect and changes in pain modulation (Wiech and Tracey, 2009) as opposed to a specific pain symptom. However, the direction of the gray matter abnormalities in other brain regions varies across patient populations. For example, S1 shows thickening in patients with TMD (Moayedi et al., 2011), but shows thinning in patients with trigeminal neuropathic pain compared to controls (Gustin et al., 2011). Differences in the direction of abnormality may reflect specific symptomology (e.g., hyper- vs. hypo-sensitivity), as it is following peripheral nerve injury (Taylor et al., 2009) and provide insight into disease mechanisms. For example, mechanisms pertaining to gray matter underlying MR-detectable differences in brain structure may include changes in neuronal size or number, synaptogenesis, dendritic branching, axon sprouting, synaptic pruning, neuronal cell death, alterations in vasculature and the size or numbers of glial cells (Blumenfeld-Katzir et al., 2011; Zatorre et al., 2012).

**Figure 4:**
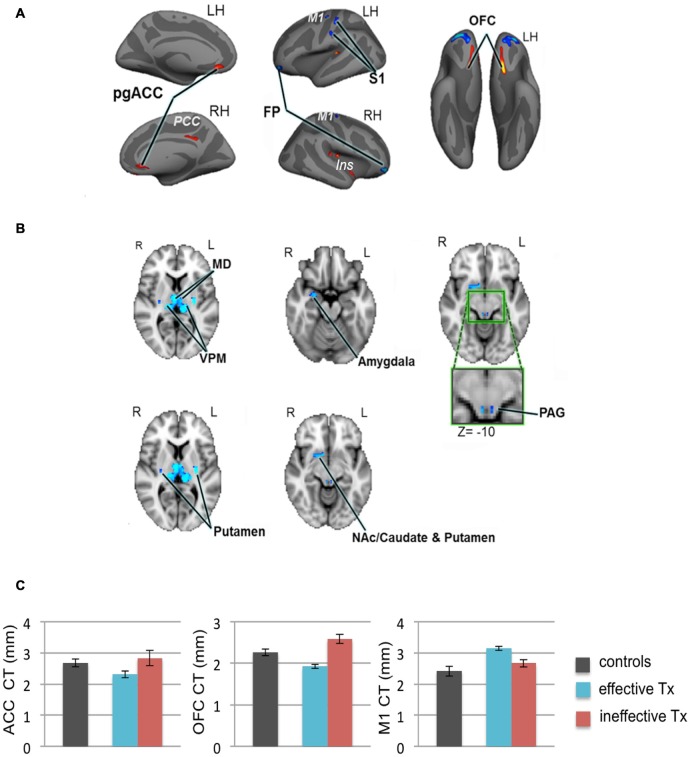
**Widespread gray matter abnormalities in patients with TN. (A)** CT analysis revealed several cortical gray matter abnormalities in TN patients. Compared to controls, patients had thinner cortex (red clusters) in the pgACC bilaterally, the right ins including the dorsal posterior insula and the ventral anterior insula (vAI), and the ventral OFC bilaterally. TN patients had thicker cortex (blue clusters) in the bilateral FP and M1, and left S1 (putative face area, contralateral to their side of pain). **(B)** Patients also had several subcortical gray matter abnormalities as determined using voxel-based morphometry. Compared to controls, TN patient had larger volumes (blue clusters) in the sensory thalamus, including the MD and VPM thalamus bilaterally, the right amygdala, the posterior putamen bilaterally, PAG (green box shows magnified region), and regions of the right NAc, anterior putamen, and caudate. **(C)** Prior to surgery, CT abnormalities (compared to healthy controls) in the pgACC, the OFC and M1, were more pronounced in the TN patients who had significant pain relief after effective Tx (blue bars) compared to those in which treatment was ineffective (red bars). These pre-treatment baseline CT levels were significantly different between the groups of patients that achieved effective vs. ineffective effects of surgery. While it remains unknown as to whether these abnormalities are a consequence of the pain or are pre-existing and contribute to the development of TN, these preliminary data suggest that certain abnormalities may have predictive value to identify patients that will benefit from treatment. Abbreviations: LH, left hemisphere; RH, right hemisphere; R, right; L, left; pgACC, pregenual anterior cingulate cortex; PCC, posterior cingulate cortex; ins, insular cortex; OFC, orbitofrontal cortex; FP, frontal pole; M1, primary motor cortex; S1, primary somatosensory cortex. MD, medial dorsal nucleus (thalamus); VPM, ventral posterior medial nucleus (thalamus); NAc, nucleus accumbens; PAG, periaqueductal gray. CT, cortical thickness; Tx, treatment. Panels **(A,B)** have been reproduced from DeSouza et al. (2013); © 2013 DeSouza et al.

We recently employed high-resolution T1-weighted scans of patients with TN and examined subcortical and cortical brain gray matter using voxel-based morphometry and CT analyses, respectively (DeSouza et al., 2013). Compared to healthy controls, we identified widespread abnormalities in regions that contribute to sensory-discriminative and cognitive-affective dimensions of pain, pain modulation, and motor function. Specifically, TN patients had greater gray matter in the thalamus, contralateral S1 (putative face area), amygdala, frontal pole (FP), periaqueductal gray (PAG), primary motor cortex (M1) and basal ganglia, and cortical thinning in the orbitofrontal cortex (OFC), pregenual anterior cingulate cortex (pgACC) and insula (DeSouza et al., 2013; Figures 4A,B). These findings may reflect unique symptomatology because TN is characterized by paroxysmal pain triggered by innocuous stimuli or movements, and is without major sensory loss. The findings may be a consequence of the pain or be pre-existing and contribute to the development of TN (Figure 4C).

A handful of other studies have examined gray matter in patients with trigeminal pain syndromes including TN. Similar to our findings, less gray matter in the ACC, anterior insula, and OFC were reported in these patients compared to healthy controls (Gustin et al., 2011; Obermann et al., 2013; Li et al., 2016). However, in contrast to our findings, the patients also had decreased gray matter in the S1, thalamus (Gustin et al., 2011; Obermann et al., 2013) and caudate nucleus. The differences in these findings with our own may be due to the mixed trigeminal pain patient groups that were examined and the different methodology used to assess gray matter. For example, in one study, the patient group included those with either trigeminal neuropathic pain or TN (Gustin et al., 2011). In this study, the authors reported that only patients with trigeminal neuropathic pain (and not patients with TN) contributed to the decreased thalamic volume result in patients. In another study, patients with classical TN and TN with concomitant facial pain were compared to healthy controls (Obermann et al., 2013). While the authors report no differences between the two patient populations, the results reported derive from a mix of both types of trigeminal pain compared to controls. Additionally, the use of different methods to assess cortical gray matter may have contributed to the differences in results. The human cerebral cortex of humans is highly folded with its thickness varying across regions. CT analysis (as implemented by DeSouza et al., 2013) was developed to provide an automated means to accurately measure the thickness of the cerebral cortex with submillimeter accuracy. In contrast, voxel-based morphometry approaches (as implemented by Gustin et al., 2011; Obermann et al., 2013; Li et al., 2016) are less accurate or can be insufficient in detecting cortical differences (Fischl et al., 2004).

**Figure 5:**
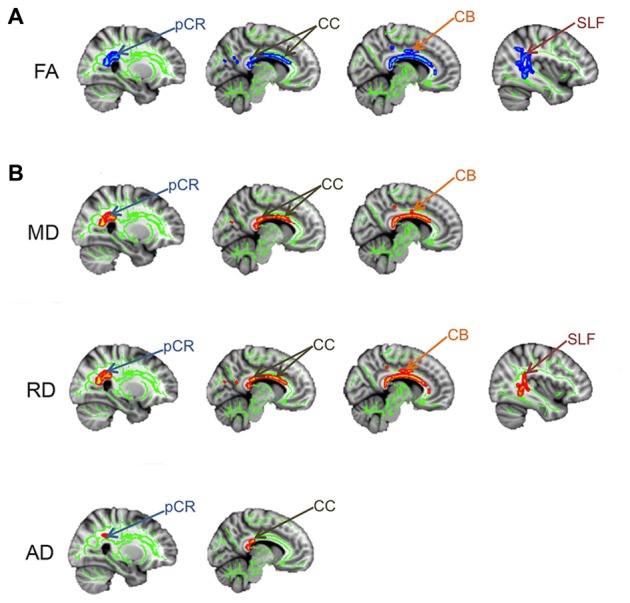
**Brain white matter abnormalities in TN.** Tract-based spatial statistics results showing that TN patients have several regions of abnormal brain white matter, compared to controls. Specifically, TN patients had significantly lower FA (blues clusters; **A**) and higher MD, RD and AD (red clusters; **B**) in the white matter areas indicated by arrows**.** pCR, posterior corona radiata; CC, corpus callosum; CB, cingulum bundle; SLF, superior longitudinal fasciculus; FA, fractional anisotropy; RD, radial diffusivity; MD, mean diffusivity; AD, axial diffusivity. This figure has been reproduced from DeSouza et al. (2014) with permission (RightsLink license number: 3823931416268).

In addition to widespread gray matter abnormalities, several studies have reported abnormalities in the brain white matter of chronic pain patients, many of which interconnect gray matter regions involved in the multi-dimensional experience of pain. A popular method used to examine white matter abnormalities in chronic pain patients compared to healthy controls is Tract-Based Spatial Statistics, which makes whole-brain comparisons of white matter DTI metrics (Rocca et al., 2008; Moayedi et al., 2012; Szabó et al., 2012). White matter abnormalities in the form of lower FA have been reported in several chronic pain disorders including migraine (Szabó et al., 2012) and TMD (Moayedi et al., 2012). The location of these abnormalities is variable between chronic pains and may be related to factors such as disease duration or pain symptomology (Rocca et al., 2008). To determine if there is abnormal brain white matter in patients with TN, we compared brain FA between TN and healthy control groups using Tract-Based Spatial Statistics (DeSouza et al., 2014). We found that patients had widespread abnormalities characterized by lower FA in the corpus callosum (CC), cingulum, posterior corona radiate (pCR) and superior longitudinal fasciculus (SLF) contralateral to their side of pain (Figure 5A). Within these regions of lower FA, we also determined that RD and MD were significantly higher in the CC and cingulum (with AD also being higher in the splenium of the CC), and that RD was higher in the SLF (Figure 5B). These tracts connect some gray matter regions involved in the multi-dimensional experience of pain, attention, and motor functions that we previously showed to be abnormal in TN. Therefore, while TN is known to involve dysfunction of the trigeminal nerve, patients also have several areas of abnormal brain white matter that may be contributing to the unique symptoms of TN pain and/or compensatory motor behaviors. In combination with the gray matter findings, these results suggest a role for the CNS in the development and/or maintenance of TN.

While in these cross-sectional studies it was not possible to confirm whether the observed brain abnormalities were pre-existing or occurred as a consequence of TN, studies examining how effective treatment impacts these abnormalities are starting to provide valuable insight.

## Treatment Effects on MR-Detectable Abnormalities in TN

**Figure 6:**
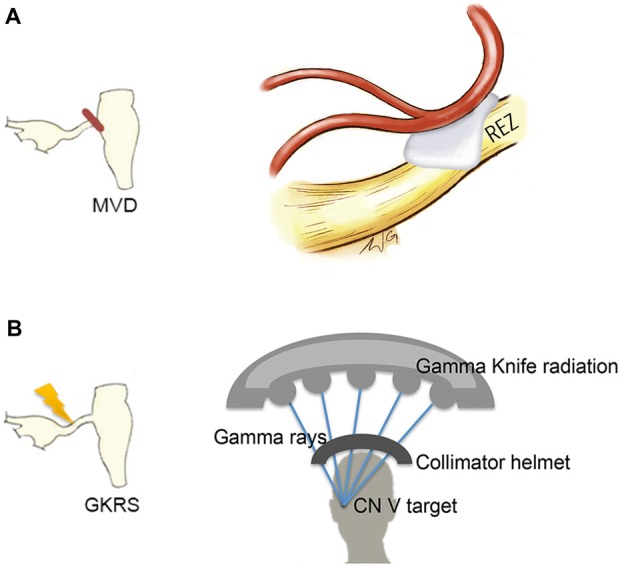
**Neurosurgical treatment options for TN.** Two neurosurgical treatment options for TN include microvascular decompression (MVD) surgery, which aims to decompress the trigeminal nerve at the REZ **(A, left)** and Gamma Knife radiosurgery (GKRS), which targets the trigeminal nerve distal to the REZ **(B, left)**. MVD surgery uses a retrosigmoid craniotomy to remove a small piece of skull via a small incision behind the ear. The cerebellopontine angle is then approached, and using microscopic magnification, the location of the trigeminal nerve and offending vessel is identified. Nerve decompression is done by dissecting the arachnoid tissue surrounding the nerve and offending vessel and placing pieces of shredded Teflon© between them **(A, right)**. GKRS is a focal radiosurgery technique. Gamma rays are emitted from approximately 200 radioactive cobalt-60 sources precisely focused on the trigeminal nerve using a collimator helmet **(B, right)** and a stereotactic frame (not shown). Abbreviations: CN V, trigeminal nerve; REZ, root entry zone.

**Figure 7:**
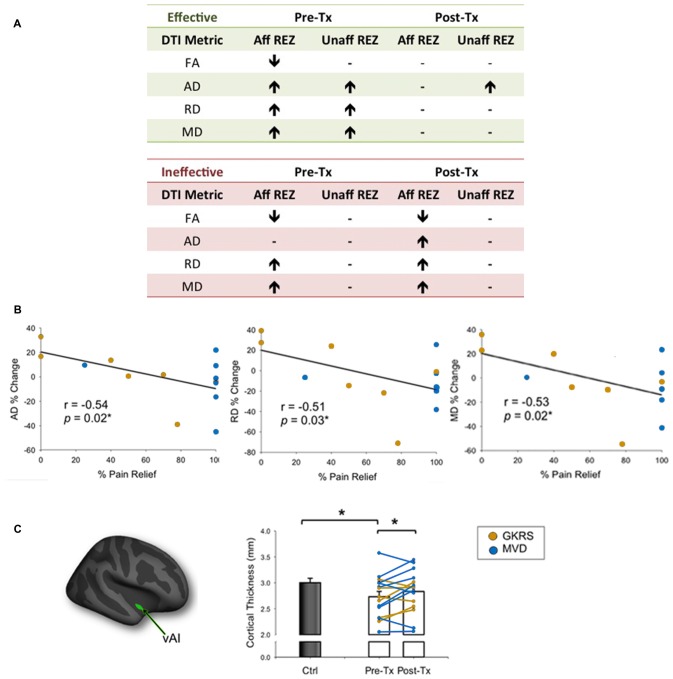
**Effects of neurosurgical treatment for TN on trigeminal REZ and brain abnormalities (A).** Patients undergoing neurosurgical treatment for TN were deemed to have either effective or ineffective treatment based on a threshold of ≥75% reduction in baseline pain intensity ratings. Only patients that had effective treatment showed a reversal of pre-treatment trigeminal REZ abnormalities in FA, AD, RD and MD following treatment (green box). In contrast, pre-treatment REZ abnormalities persisted in patients that had ineffective treatment (green box). Arrows indicate direction of abnormalities relative to controls. **(B)** The degree of normalization of AD, RD and MD abnormalities only at the affected trigeminal REZ was associated with the degree of pain relief achieved by TN patients. **(C)** The vAI (green cluster on inflated brain) was the only gray matter region to normalize in patients following effective treatment only. Specifically, prior to treatment patients had significantly thinner vAI thickness compared to controls. Following treatment, vAI thickness significantly increased to become comparable to control vAI thickness. Bar graphs of mean CT values ± SEM (in mm) are shown for controls (black bars) and patients (white bars). Individual patient data are also shown pre- and post-treatment, with patients who received GKRS in orange, and MVD surgery in blue. Tx, treatment; Aff REZ, affected root entry zone; Unaff REZ, unaffected root entry zone; FA, fractional anisotropy; RD, radial diffusivity; MD, mean diffusivity; AD, axial diffusivity; Ctl, controls; vAI, ventral anterior insula; GKRS, Gamma Knife Radiosurgery; MVD, microvascular decompression. Panels **(B,C)** have been adapted from DeSouza et al. (2015) with permission (Rightslink license number: 3823931191470). Asterisks indicate significance at *p* < 0.05 level.

A handful of studies have used structural MRI to demonstrate that some gray matter abnormalities in patients with chronic pain are reversible following effective treatment (Gwilym et al., 2010; Seminowicz et al., 2011; Rodriguez-Raecke et al., 2013). This suggests that they in part arise as a consequence of being in pain and do not necessarily cause the pain. There are currently several treatment approaches available for the management of TN. Although medications are always the first line of treatment (Zakrzewska and Akram, 2011), over time, pain attacks may be longer lasting, occur more frequently, and be less responsive to medications, leading patients to seek neurosurgical intervention for pain relief (Nurmikko and Eldridge, 2001). Surgical treatments for TN target the trigeminal nerve at different anatomical sites (Figure 6) with the goal of displacing the offending vessel at the trigeminal REZ, as in microvascular decompression (MVD) surgery, or partially injuring the trigeminal nerve distal to the REZ to reduce nociceptive signaling, as in Gamma Knife radiosurgery (GKRS; Figure 6; Zakrzewska and Akram, 2011). Both of these treatments result in a high proportion of patients with good or complete pain relief. As such, we recently examined how effective MVD surgery, and GKRS (as denoted by a 75% reduction in pre-treatment pain intensity scores) impact brain gray matter and trigeminal REZ abnormalities as measured by T1-weighted and DTI images, respectively (DeSouza et al., 2015). Our results indicated that effective surgical treatment was associated with the resolution of trigeminal abnormalities at both the affected and unaffected REZ (Figure 7A). However, only the proportion of MD, RD and AD normalization at the affected REZ correlated with the degree of pain relief (DeSouza et al., 2015; Figure 7B). These DTI findings at the REZ support a peripheral theory of TN and corroborate the importance of the REZ in TN pathophysiology.

In addition to the REZ normalization, we also found that right ventral anterior insula (vAI) thinning normalized towards the level of healthy controls following effective neurosurgical treatment for TN (DeSouza et al., 2015; Figure 7C). The right-lateralization of this finding is consistent with other studies that have reported right-lateralized activations in the context of pain expectation and the emotional modulation of pain (Wiech and Tracey, 2009). Interestingly, vAI normalization was not associated with pain relief as shown in other chronic pain disorders (Seminowicz et al., 2011), suggesting that for TN patients, vAI normalization may have occurred secondarily to the resolution of the affected REZ abnormalities, which were associated with pain relief. Since most studies of chronic pain report thinning of the AI, cingulate, and prefrontal cortices, it is possible that these abnormalities reflect pain chronicity in general, or other aspects of pain perception such as negative affect (Wiech and Tracey, 2009; Kurth et al., 2010; Chang et al., 2013). Since effective treatment can reverse vAI thinning in TN, our study provided novel evidence that some central abnormalities occur as a consequence of TN pain and likely contribute to the maintenance of this disorder.

## Predicting TN Treatment Outcomes: The Future of Imaging?

While many patients experience excellent outcomes from TN treatments, some experience minimal pain relief. That is, there is individual variability with regard to patient responsiveness to treatment. Since conventional clinical diagnostic measures cannot predict which patients will benefit from a given treatment approach for TN, neuroimaging may be an effective tool to noninvasively provide brain-based biomarkers to support the optimal treatment selection for individual TN patients. We previously established that TN is associated with subtle, yet widespread abnormalities in brain structure as measured by morphometric and microstructural measures of brain gray matter and white matter (DeSouza et al., 2013, 2014). Furthermore, our preliminary data suggest that some pre-treatment cortical abnormalities differ between patients that achieve good vs. poor treatment outcomes (Figure 4C). As such, future studies may determine which of these measures most accurately predict treatment outcome using a machine learning approach. The application of machine learning algorithms to pain neuroimaging has become a recent topic of interest (e.g., Woo et al., 2014; Chong et al., 2016; Lindquist et al., 2016; Tu et al., 2016), with clinical applications arguably having the greatest need for this type of prediction method (Rosa and Seymour, 2014).

Machine learning approaches based on neuroimaging data have been used to distinguish physical and social pain/rejection (Woo et al., 2014), differentiate migraine patients from controls, and decode subjective pain intensities (Wager et al., 2013; Tu et al., 2016). In general, neuroimaging data can be used to train a discriminative classifier, such as a support vector machine, to make predictions about new data. Clinically, it would be highly advantageous to have brain-based biomarkers that can predict outcomes in individual patients based on models that were trained on prior groups to make out-of-sample predictions (Lindquist et al., 2016). Knowing in advance which patients may better benefit from a certain treatment type (e.g., GKRS) would be enormously important for personalized and effective pain management, and it would reduce costs associated with unsuccessful treatment.

While the predictive accuracy of a classification algorithm based solely on morphological measures is cautioned (Labus et al., 2015), structural MRI measures in combination with functional connectivity measures may be highly informative for predicting treatment response. In one study, a combination of structural and resting-state functional MRI measures were shown to accurately predict treatment response in patients with social anxiety disorder (Whitfield-Gabrieli et al., 2015). It could also provide insight into the fundamental mechanisms underlying individuality of pain and its alleviation and complement patient self-reports.

## Summary

In this review, we have provided structural neuroimaging evidence to support peripheral theories of TN etiology and pathophysiology, showed a role for the CNS in TN pain, and discussed implications for the use of potential neuroimaging-based biomarkers to predict treatment response. It is becoming increasingly understood that TN pathophysiology is associated with both trigeminal nerve and brain abnormalities. While future studies are needed to assess the extent to which these abnormalities are the cause or consequence of TN, it is clear that structural MRI methods offer a valuable means to noninvasively examine the trigeminal system *in vivo* and inform our understanding of trigeminal nerve and brain abnormalities in TN.

## Author Contributions

DDD: concept and design for manuscript content; critical revision of the manuscript for important intellectual content. MH and KDD: concept and design for manuscript content; interpretation of findings; critical revision of the manuscript for important intellectual content; supervising.

## Conflict of Interest Statement

DDD reports no disclosures. MH is the surgical co-director of the Gamma Knife Centre at the Toronto Western Hospital and has received financial contributions from the Canadian Institutes of Health Research and the Trigeminal Neuralgia Association of Canada. KDD reports no disclosures.
